# Physician’s Visiting List for 1896

**Published:** 1895-12

**Authors:** 


PHYSICIANS' VISITING LIST FOR 1896: Lindsay & Blakiston.

This well-known Visiting List, published annually for forty-five years, presents several improvements in the new edition for 1896.

More space has been allowed for writing the names and to the "Memoranda Page;" a column has been added for the "Amount" of the weekly visits and a column for the "Ledger Page."

To do this without increasing the bulk or price, the reading matter and memoranda pages have been rearranged and simplified.

The lists for 75 patients and 100 patients will also have special memoranda page as above, and hereafter will come in two volumes only, dated January to June and July to December. While this makes a book better suited to the pocket, the chief advantage is that it does away with the risk of losing the accounts of a whole year should the book be mislaid.

The publishers announce that before making these changes they have personally consulted a number of physicians who have used the book for many years, and have taken into consideration many suggestions made in letters from all parts of the country.

No Visiting List has been used to such an extent or for so long a time as this. There is none better suited to the work of the general physician in keeping easily and systematically his business accounts and memoranda.

